# Post operative sore throat: Comparison between Macintosh versus Video Laryngoscope in patients intubated by trainee anaesthetists - A Randomised Control Trial

**DOI:** 10.12669/pjms.37.3.3365

**Published:** 2021

**Authors:** Amin Ahmed Kapadia, Faraz Shafiq, Amir Raza

**Affiliations:** 1 Amin Ahmed Kapadia Senior Medical Officer, Department of Anaesthesiology, Aga Khan University, Karachi, Pakistan; 2 Faraz Shafiq Assistant Professor, Department of Anaesthesiology, Aga Khan University, Karachi, Pakistan; 3 Amir Raza Statistician, Department of Anaesthesiology, Aga Khan University, Karachi, Pakistan

**Keywords:** Sore throat, Laryngoscopes, Anesthetists

## Abstract

**Objectives:**

Postoperative sore throat (POST) is a common complication related to endotracheal intubation. The aim of this study was to compare the incidence of POST in patients intubated by trainee anaesthetist using Video Laryngoscope™ (VDL) or Conventional Macintosh Laryngoscope (CL).

**Methods:**

Total 110 patient scheduled for elective laparoscopic cholecystectomy were included from main operating room of Aga Khan University Hospital between June 2017-2018. The standardized perioperative protocol was used for general anaesthesia. Selected patients were randomly allocated into conventional laryngoscopy (CL) group or video laryngoscopy (VDL) group. The evaluation of sore throat was done at 1st, 12th and 24th hour postoperatively using a ten-point visual analogue scale.

**Results:**

The demographic characteristics, including intubation time, related complications or any other maneuver required were similar between the groups. The incidence of POST at 1st hour was 47% patients in CL group and 38% in VDL group (p=0.335). At 12th hour, 34.5% patients in CL and 38% in VDL reported POST (p=0.692). Similarly at 24th hour, 25% patients in CL and 16% in VDL group reported POST (p=0.669).

**Conclusions:**

There was no significant difference in incidence of POST for patients intubated by trainee anaesthetists using either CL or VDL. Objective evidence of training and laryngoscope technique can impact of POST.

## INTRODUCTION

Airway management is a vital aspect of maintaining oxygenation and ventilation during General anaesthesia (GA). The technique requires laryngoscope-guided insertion of the tracheal tube, which needs to be passing down beyond the vocal cords. The procedure is not risk free and has associated complications.^1^ The early physiological effects of intubation might be presented in form of hemodynamic fluctuations under GA.^2^ While, complications like post-operative sore throat (POST) evident during recovery phase.^3^ The reported incidence of POST is somewhere between 30-70%.^4^ The trauma related to intubation is one of the main factor behind the occurrence of POST. A VDL™ (C-MAC Karl Storz Video Laryngoscope) works like the conventional laryngoscope. However, having camera at its tip is the key feature offers advantage of live video streaming on to the monitor. This enables operator, as well as persons around to visualize glottic view. Studies have proven that this arrangement improves number of intubation attempts, difficulty and also reduces the complications related to intubation.^5^

Studies also have shown the likelihood of lesser force of impact in comparison to conventional Macintosh laryngoscope, which might lower, the incidence of POST in patients managed with VDL.^6^ In most of these recent studies experienced anaesthetists mainly did intubations in studies protocol. However, if its usage by trainee anaesthetist is associated with any improvement in the incidence of POST, we don’t know. The objective of this randomized control trial was to compare the incidence of POST in patients intubated by trainee anaesthetist using conventional or Video Laryngoscope.

## METHODS

The study protocol was approved from Ethical Review Committee, Aga Khan University (4750-Ane-ERC-17). The trial was also registered at www.clinicaltrials.gov. (Identifier: NCT04334616). The study was conducted in the main operating room Aga Khan University for a period of one year after approval of study on 19^th^ June 2017. (June 2017-2018) All adult patients of age between 20-60-year, American Society of Anaesthesiologist (ASA) Grade I and II, scheduled for elective laparoscopic cholecystectomy were enrolled in the study. Patient with anticipated difficult airway as assessed by limited mouth opening (< 2 finger breadth), limited neck extension, any anatomical/pathological airway abnormality or history of radiotherapy in head and neck region were excluded from study. Similarly, obese patients having BMI>30 kg/m^2^, history of gastro esophageal disease (GERD) requiring rapid sequence induction with cricoid pressure, and those who were not able to intubated within three laryngoscopy attempts were excluded from study protocol.

After taking informed consent selected patients were randomly allocated by a computer-generated number, either into conventional laryngoscopy (CL) group or video laryngoscopy (VDL) group using sealed envelope technique. All patients were managed with GA, requiring control mode ventilation and intubation under supervision of consultant anaesthetist.

After instituting routine ASA recommended monitoring standards, induction of anaesthesia was done using Propofol 1.5-2 mg/Kg, Nalbuphine 0.1 milligram per Kg and Atracurium 0.5 mg/Kg. The readiness of intubating condition was judged by orbicular oculi response to train of four stimuli. Patients were considered ready to intubate when there was no response to the neuromuscular stimuli. The sniffing position with under head pillow was maintained to facilitate all intubations. Selection of VDL or CL group was done as per randomization. Anesthesia resident Level I and II having experience of more than six months did all intubations. Patient in CL were intubated with laryngoscope size three or four blade. Similarly, patients in VDL were intubated by size three or four blade as per decided plan. Appropriate size of endotracheal tube (ETT) 7-7.5 mm ID was used for adult females, while male patients required size 8-8.5 mm ID of ETT. The ETT tubes were lubricated with water-based gel (Aplicare Lubricating Jelly) before insertion.

The intubation time if less than or greater than 30 seconds, including number of intubation attempts, alternate maneuver used, complications like dental, oral mucosal trauma or blood on laryngoscope were recorded. The inflation of cuff was guided by any obvious leak as measured by the adjustable pressure limiting valve (APL) bag valve pressure of 20 mm Hg. Later on, inflation of cuff pressure was also confirmed by pressure manometer. The cuff pressure was maintained between 20-25 mm Hg.

Orogastric (OG) tube lubricated by water-based gel was inserted in all patients. Number of attempts in passing OGtube, or if required any need of Magill forceps was also noted. All patients were positioned supine initially and then reverse trendelenburg, which is demand for laparoscopic cholecystectomy. Anesthesia was maintained with Isoflurane in mixture of O_2_/Air. Dual antiemetic prophylaxis. Dexamethasone 0.1 mg/Kg at start and Ondansteron 0.1 mg/Kg at the end was used for every patient. After completion of surgical procedure, the paralytic effect of Atracurium was assessed using twitch response to train of four stimuli. All patients were extubated as guided by the subjective and objective criteria of extubation. Patients were observed for POST at 1^st^, 12^th^ and 24^th^ hours post operatively by the primary investigator who was blinded to allocated group. The visual analogue scale (0-10) was used to evaluate the severity of POST.

### Statistical Analysis:

Sample size calculation was based on primary outcome i.e. incidence of POST. Najafi A et al.^7^ reported the incidence of POST 0.28 and 0.54 in VDL and CL Group. A total of 55 patients in each group was needed to achieve 20% reduction in POST with 80% power and 5% Type-I error. Primary outcome was POST. CL or VDL was taken as intervention. Normally distributed point estimation was reported in term of mean and standard deviation. Student’s t test was used to analyze the difference between two groups for age, weight, height and body mass index (BMI). Frequency and percentages were computed for variables like gender, Mallampatti grade, size of laryngoscope, alteration of airway management, Cormack & Lehane intubation grade, number of laryngoscopy attempts, associated complication and POST. All were analyzed by Chi-square test or fisher exact test. For multivariate analysis POST was converted into binary outcome (VAS; None = 0 and mild/moderate/severe considered as one). Effect of the intervention on POST overtime (measured three times at one 12 and 24 hours post operatively) was assessed using a generalized estimating equations (GEE) with categorical time as within subject variable, auto-regressive (AR1) working correlation matrix structure by using logistic regression model. Main effects were time and intervention. Interaction between time and intervention was tested. Odds ratio with 95% confidence were reported. All analysis was performed with statistical packages for social science version 19 (IBM SPSS Inc., Chicago, IL, USA). The P ≤ 0.05 was considered as significant.

## RESULTS

Total 110 patients, 55 in each group were included. All participants were able to follow study protocol. The demographic data of patients showed no difference in characteristics like age, gender, weight, height, BMI, ASA classification, and Malampatti scoring amongst the group (Table-I).

**Table-I:** Demographic characteristic between both groups.

Variables	CL (n=55)	VDL (n=55)	P-Value
Age (Years)	42.4±13.72	42.13±12.69	0.914
Weight (kg)	67.38±12.52	71.15±12.29	0.118
Height (cm)	160.85±10.71	159.66±8.98	0.529
BMI (kg/m^2^)	25.74±3.73	27.46±4.09	0.023
***Gender***			
Male	18(32.7%)	15(27.3%)	0.533
Female	37(67.3%)	40(72.7%)	
***ASA Status***			
I	21(38.2%)	22(40%)	0.845
II	22(61.8%)	33(60%)	
***Mallampatti Grade***			
I	28(50.9%)	23(41.8%)	
II	22(40%)	26(47.3%)	0.633
III	5(9.1%)	6(10.9%)	

Similarly, the time required to intubate, intubation grade, number of patients who were intubated in first attempt and those who require bougie or any other airway maneuver for intubation were also similar between both groups (Table-II).

**Table-II:** Airway management between the groups.

Variables	CL (n=55)	VDL (n=55)	P-Value
***Blade Size***			
3	46(83.6%)	51(92.7%)	0.140
4	9(16.4%)	4(7.3%)	
Alteration of technique	8(14.5%)	5(9.1%)	0.376
BURP	8(14.5%)	6(10.9%)	0.567
Required Bougie	4(7.3%)	6(10.9%)	0.507
***Cormack & Lehane grade***			
I	45(81.8%)	52(94.2%)	0.039
II	10(18.2%)	3(5.5%)	
***Number Laryngoscopy attempts***			
I	50(90.9%)	51(92.7%)	0.926
II	4(7.3%)	3(5.5%)	
III	1(1.8%)	1(1.8%)	
***Duration of Intubation***			
≤ 30Sec	46(83.6%)	44(80%)	0.621
> 30Sec	9(16.4%)	11(20%)

The study was done in patients having laparoscopic cholecystectomy, which requires OG tube insertion. The number of attempts requires to insert OG tube or need of using Magill’s forceps was also similar between the groups. Procedural complications related to endotracheal intubation included blood on the laryngoscope, dental trauma and soft tissue damage were also similar between both groups (Table-III).

**Table-III:** Comparison between number of attempts required for passing OG tube, Use of Magill’s forceps and overall complications between the groups.

Variables	CL (n=55)	VDL (n=55)	P-Value
***Number of attempts required for passing OG Tube***			
1	39(70.9%)	40(72.7%)	
2	11(20%)	14(25.5%)	0.219
3	5(9.1%)	1(1.8%)	
Use of Magill’s Forceps for passing OG tube	5(9.1%)	3(5.5%)	0.463
***Complications***			
Soft Tissue Damage	7(12.7%)	6(10.9%)	0.768
Teeth Injury	1(1.8%)	1(1.8%)	0.999
Blood on Laryngoscope	2(3.6%)	1(1.8%)	0.558

**Fig.1:**
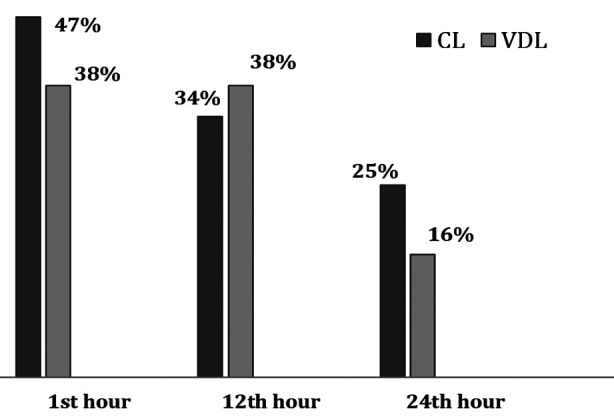
Incidence of POST at various time intervals.

The comparison related to incidence of POST at various time intervals is shown in (Fig.1). At first hour, 47% patients in CL group and 38% in VDL group reported POST. Estimated POST difference between groups was not signficant [difference of 9%; 95%CI: -9.4% to 27.4%, p=0.335]. At twelth hours, 34.5% in CL and 38% in VDL reported POST. The diference was again nonsignficant [difference of 3.6%; 95%CI: -14.3% to 21.6%, p=0.692]. Similarly at twenty forth hour, 25% patients in CL and 16% in VDL group reported POST. The difference was not signfigcant betrween groups [difference 3.6%; 95%CI: -13.0% to 20.3%, p=0.669]. The severity of POST as measured by VAS, at various time intervals is mentioned in (Table-IV). This again shows non significant difference amonst the group.

**Table-IV:** Comparison of severity of POST between groups.

Time	CL (n=55)	VDL (n=55)	P-Value
***(1^st^ Hour)***			
Mild	15(27.3%)	10(18.2%)	
Moderate	10(18.2%)	11(20%)	0.485
Severe	1(1.8%)	0(0%)	
***(12^th^ Hour)***			
Mild	13(23.6%)	15(27.3%)	
Moderate	6(10.9%)	6(10.9%)	0.905
Severe	0(0%)	0(0%)	
***(24^th^ hours)***			
Mild	10(18.2%)	15(27.3%)	
Moderate	4(7.3%)	1(1.8%)	0.241
Severe	0(0%)	0(0%)	

Longitudinal analysis uisng GEEs, demonstrate that main effect of intervention, categorical time and number of attempts of passing orogastric tube were not significant however intervention and time interaction effect was observed significant as shown in (Table-V).

**Table-V:** Analysis of the GEE parameter estimates with auto regressive working correlation with POST as outcome variables.

Predictors	Estimated(SE)	P-Value	OR[95%CI]
***Groups***			
CL	0.079(0.40)	0.85	1.08[0.62-1.78]
VDL	Ref		
***Time***			
24h	-0.41(0.21)	0.06	0.66[0.43-0.1.01]
12h	-0.002(0.112)	0.98	0.99[0.80-1.24]
1h	Ref		
***Number of attempts required for OG tube***			
1	-1.11(0.81)	0.17	0.33[0.07-1.65]
2	-0.85(0.89)	0.33	0.42[0.07-2.44]
3	Ref		
***Intubation grade***			
I	-1.37(0.61)	0.025	0.25[0.08-0.84]
II	Ref		
Group * Time			
Group CL * 24 hours	-0.74(0.37)	0.049	0.48[0.23-0.99]
Group CL * 12 hours	-0.66(0.23)	0.006	0.52[0.33-0.83]
Group CL * 1 hours	Ref		
Intercepts	1.82(1.04)	0.08	

## DISCUSSION

POST has been a well-recognized complication after GA. Though it is a minor side effect, but associated with significant dissatisfaction from the patient side.^8^ Our study did not show any advantage of VDL in this scenario, where trainee anaesthetists used it. However, the overall incidence is low as compared to various reported studies in literature. The study conducted by Lee JY et al.^9^ in Korean population revealed the overall incidence of 57.5%. In their study, all intubations (n=221) were done primarily by two experienced anaesthetists. While in our local population it was found to be around 26%, undergoing different type of general and gynecologic surgeries.^10^

In contrast, trainees exclusively did intubations in our study population undergoing elective laparoscopic cholecystectomy. Studies incorporating VDL techniques revealed better results with these devices. This is mainly because of impact of lesser force on oropharyngeal structure. This could be added advantage if used by trainees.^11^

A cochrane review by Lewis SR et al.,^12^ showed similar benefit of VDL in terms of providing better glottic view, reducing the number of intubation attempts and hence trauma associated with laryngoscopy. However, the results were not convincing in reducing incidence of POST and there was wide variation amongst surgical procedures. The major strength of our study protocol is standardization of surgical procedure, that is Laparoscopic Cholecystectomy. This was planned to provide similar perioperative condition including the positioning and duration of anaesthesia. They all can influence the outcome related to POST. Studies have also mentioned the impact of applied force during intubation on associated trauma and related complications. This definitely gets aggravated in patients being intubated by trainees.^11^ As mentioned earlier, the use of VDL in this scenario is logical to overcome these issues. The learning curve is short and one can get easily trained for it. This is also reflected by our study. As after six months of initial training the procedural performance, and outcomes were similar in between groups. However, this was not reflected in terms of decreasing the incidence of POST. This may be related to the fact that no formal teaching plan was introduced for the residents to get trained with VDL. Moreover, we didn’t have any objective evidence about how many intubations they have done with either of the laryngoscope technique. Similarly, we don’t know was there any difference in the application of force during laryngoscope with both techniques? The study results by Aqil et al.^13^ shows significant improvement in POST with the use of Glide scope (GS) in comparison to conventional ML. However, anaesthetists involved in the study were well trained. They had done at least 100 intubations with each of the device before participating in the study protocol. We think, if intubations are going to be done by less skilled person, the use of either laryngoscope is associated with POST. Anderson LH et al.^14^ shared the similar finding. They did a comparison between GlideScope (GS) versus conventional Macintosh laryngoscope in morbidly obese patients. Though GS worked well in terms of providing better intubating conditions, and overcoming difficulty. However, the difference was not significant in terms of reducing POST. The possible reasons were prolongation of intubation time in GS group, secondly less trained intubating personals. If we want to gain the advantage of reducing POST using VDL in trainees, it’s better to get them familiarized with the technique first.

### Limitation of the study:

Our trainees had experience of only six months. Moreover, we didn’t have the objective evidence about how they have been trained and achieved competency with intubation before enrolling into study. Future studies should explore outcome related to POST with different laryngoscope devices after giving formal training with them.

## CONCLUSIONS

There was no significant difference in incidence of POST for patients intubated by trainee anaesthetists using either CL or VDL. Our recommendation is to incorporate training strategy before exploring the impact of laryngoscope techniques on POST.

### Author’s Contribution:

**AAK,** **FS** conceived, designed & editing of manuscript.

**AAK,** **FS** did data collection and manuscript writing.

**AR** did the statistical analysis

**AAK** takes the responsibility and is accountable for all aspects of the work in ensuring that questions related to the accuracy or integrity of any part of the work are appropriately investigated and resolved.
